# Stability and Strength of Monolayer Polymeric C_60_

**DOI:** 10.1021/acs.nanolett.2c04497

**Published:** 2023-01-11

**Authors:** Bo Peng

**Affiliations:** Theory of Condensed Matter Group, Cavendish Laboratory, University of Cambridge, J. J. Thomson Avenue, CambridgeCB3 0HE, United Kingdom

**Keywords:** density functional theory calculations, monolayer fullerene networks, mechanical stability, dynamic stability, thermodynamic stability

## Abstract

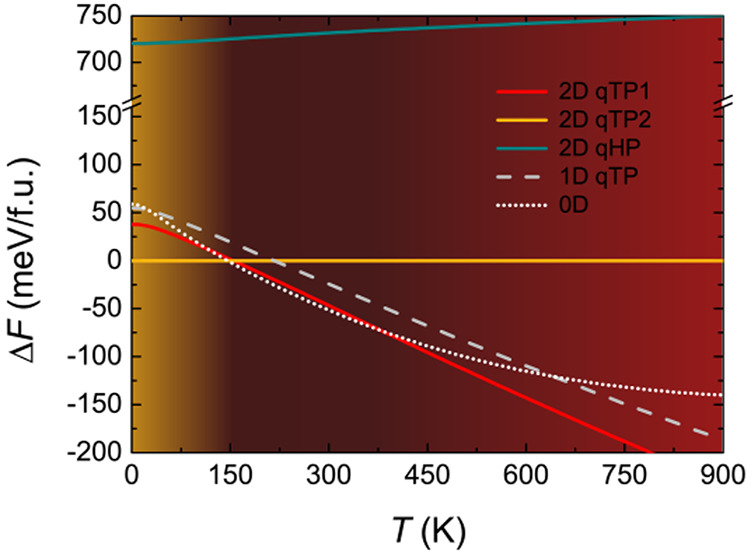

Two-dimensional fullerene networks have been synthesized in several forms, and it is unknown which monolayer form is stable under ambient conditions. Using first-principles calculations, I show that the believed stability of the quasi-tetragonal phases is challenged by mechanical, dynamic, or thermodynamic stability. For all temperatures, the quasi-hexagonal phase is thermodynamically the least stable. However, the relatively high dynamic and mechanical stabilities suggest that the quasi-hexagonal phase is intrinsically stronger than the other phases under various strains. The origin of the high stability and strength of the quasi-hexagonal phase can be attributed to the strong covalent C–C bonds that strongly hold the linked C_60_ clusters together, enabling the closely packed hexagonal network. These results rationalize the experimental observations that so far only the quasi-hexagonal phase has been exfoliated experimentally as monolayers.

Recent attempts to synthesize layers of connected buckyballs, i.e. C_60_ molecules linked by carbon–carbon bonds, have obtained different arrangements of cluster cages through the formation of bonds between neighboring C_60_ molecules.^1^ The obtained allotropes include a few-layer rectangular structure in which each C_60_ molecule has four neighboring buckyballs and a monolayer hexagonal structure in which each C_60_ cage binds to six neighbors: namely, a few-layer quasi-tetragonal phase (qTP) and a monolayer quasi-hexagonal phase (qHP), respectively. Great efforts have been devoted to stabilizing the linking bonds between neighboring cluster cages by introducing magnesium atoms to form a quasi-2D fullerene network with strong intralayer covalent bonds^1^ because Mg atoms tend to promote covalent bonds.^2,3^ To aid exfoliation, the Mg ions that hold the C_60_ cages together can be then replaced by large organic ions, which can be removed afterward by hydrogen peroxide, leading to pure, charge-neutral fullerene networks in 2D.^1,4^ Unfortunately, only qHP C_60_ has been obtained as monolayers, while all the qTP C_60_ flakes are few-layers.^1^ These results raise doubts regarding the stability of monolayer fullerene networks.

Ever since the discovery of C_60_,^5^ the formation mechanism and stability of the fullerene molecules are far from being completely understood.^6−9^ When forming structural units of C_60_ clusters in a 2D plane, it is unclear whether ordered structures of monolayer polymeric C_60_ are stable under ambient conditions such as strain and temperature. Recent first-principles calculations have investigated various structural phases of monolayer C_60_ with different bonding characters.^10−15^ The mechanical stability of several phases has been confirmed.^10,11,14^ More recently, the thermal stability of monolayer C_60_ has been addressed using molecular dynamics simulations, showing that both qTP and qHP C_60_ monolayers can remain stable at temperatures near 800 K,^16^ which is partially consistent with the experimental result that monolayer qHP C_60_ does not decompose at 600 K.^1^ However, previous analyses based on mechanical and thermal stability cannot explain why the qTP monolayers have not yet been exfoliated experimentally. Furthermore, the dynamic stability of monolayer fullerene networks with respect to lattice vibrations, which indicates whether the crystal structure is in a local minimum of the potential energy surface,^17−20^ is still unexplored. Additionally, the thermodynamic stability of different phases, which energetically classifies the stability (especially at finite temperatures),^21−33^ remains unknown. Because of such knowledge gaps, several questions need to be answered to understand the phase stability of monolayer fullerene networks. (i) Are qTP and qHP C_60_, as pure carbon monolayers without extra Mg or organic ions to bind the C_60_ cages together, dynamically stable? (ii) What is their relative stability from a thermodynamic aspect? (iii) Can the calculated mechanical strength support their phase stability?

In this work, I investigate the mechanical, dynamic, and thermodynamic stability of monolayer qTP and qHP C_60_ by using first-principles calculations. Structural relaxation obtains two crystal structures of the quasi-tetragonal phase (denoted as qTP1 and qTP2, respectively). I show that the qTP1 monolayer, albeit being thermodynamically stable at all temperatures above 380 K, possesses low dynamic and mechanical stability due to its weak bonding perpendicular to the straight chains of C_60_ buckyballs. On the other hand, although qTP2 fullerene might be the ground-state structure with the lowest Gibbs free energy at 0 K and exhibits good dynamic and mechanical stability, it is only thermodynamically stable with respect to qTP1 C_60_ at low temperatures. Instead, monolayer qHP C_60_ should be experimentally accessible due to its dynamic and mechanical stability, in spite of its lowest thermodynamic stability among all three phases. In addition, qHP C_60_ has the highest strength under various strains (hydrostatic, uniaxial, and shear) because of the closely packed crystal structures.

First-principles calculations are performed using the Vienna *ab initio* simulation package (VASP) .^34,35^ The projector augmented wave (PAW) potential is used with C 2s^2^2p^2^ valence states ,^36,37^ under the generalized gradient approximation (GGA) with the Perdew–Burke–Ernzerhof parametrization revised for solids (PBEsol) as the exchange-correlation functional.^38^ The crystal structures are optimized by fully relaxing the lattice constants and internal atomic coordination (for computational details, see the Supporting Information). Geometry optimization starting from the quasi-tetragonal phase consisting of only carbon atoms leads to a quasi-1D qTP monolayer (qTP1), as shown in Figure 1a. On the other hand, a two-step structural relaxation, starting from monolayer qTP Mg_2_C_60_ and then removing the Mg ions before the second relaxation, obtains a tightly bound qTP monolayer (qTP2), as shown in Figure 1b. The two-step structure relaxation mimics the experimental procedure to remove the charged ions introduced during synthesis.^1,4^ The computed lattice constants for all three phases are given in Table 1, which are in good agreement with previous results,^10,11,15^ therefore confirming the reliability of the present calculations.

**Figure 1:**
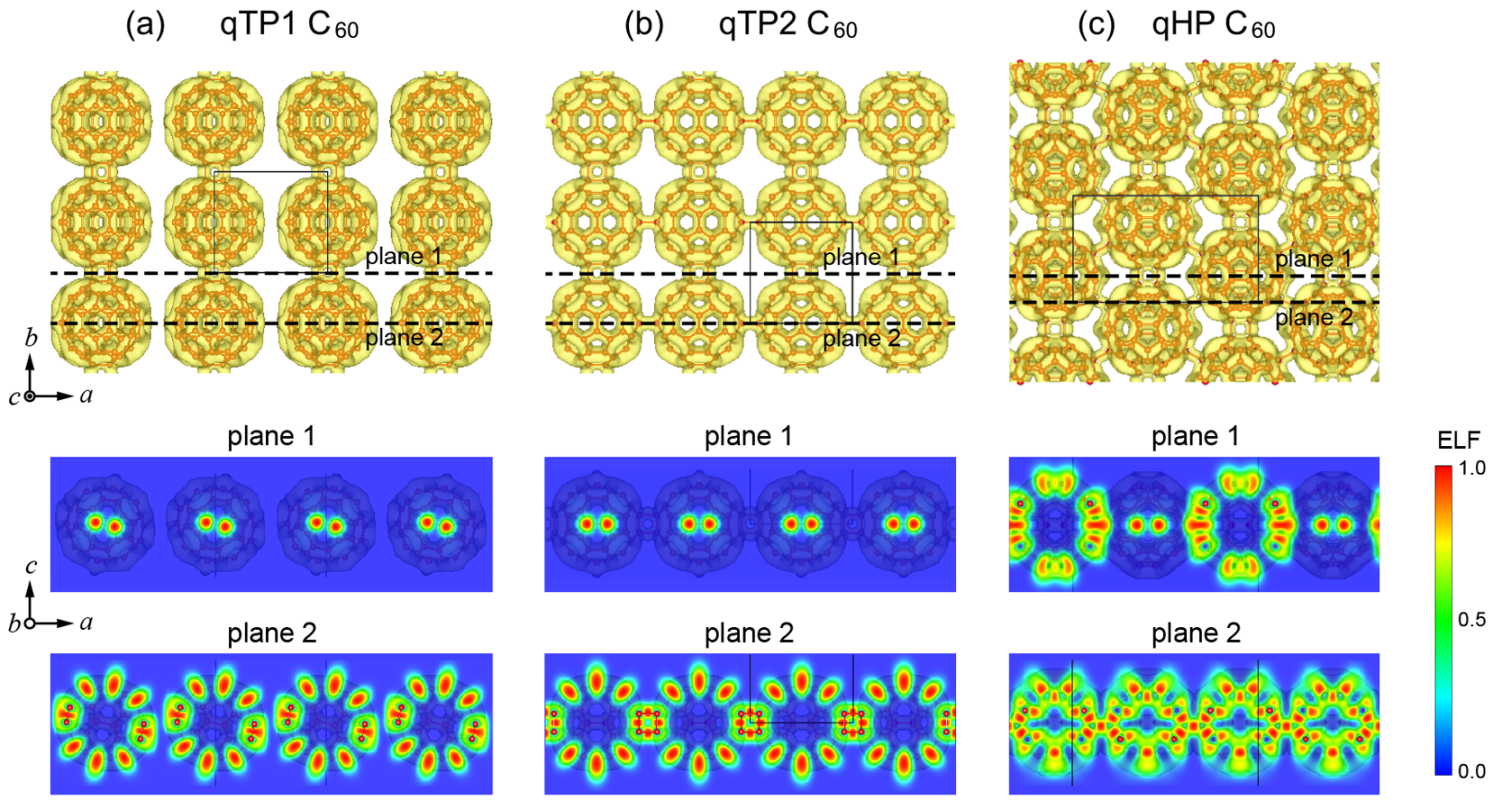
Bond structures for (a) qTP1, (b) qTP2, and (c) qHP C_60_. The default isosurface level in VESTA^39^ is used. Maps of the ELF on the (010) plane are also present.

**Table 1:** Calculated Static Lattice Constants (in Å) and Cohesive Energies *E*_c_ (in eV/atom) of qTP1, qTP2, and qHP C_60_ Monolayers, 1D qTP C_60_ Chain, and 0D C_60_ Molecule^a^

phase	*a*	*b*	*E*_c_
2D qTP1	10.491	9.063	–9.2582
	(10.522)	(9.090)	
2D qTP2	9.097	9.001	–9.2587
	(9.132)	(9.031)	
2D qHP	15.848	9.131	–9.2465
	(15.896)	(9.162)	
1D	9.062		–9.2579
	(9.098)		
0D			–9.2564

a The cohesive energy is defined as *E*_c_ = *E*_tot_/*N* – *E*_isolated_, where *E*_tot_ is the total energy of the crystal, *N* is the number of atoms in the unit cell, and *E*_isolated_ is the total energy of an isolated carbon atom. The room-temperature lattice constants calculated under the quasi-harmonic approximation are also shown in parentheses for comparison.

The bond structures at equilibrium are examined in Figure 1. The relaxed structure for qTP1 fullerene can be regarded as one-dimensional chains of C_60_ cages along the *b* direction that are linked by the nearly in-plane [2 + 2] cycloaddition bonds. In comparison, qTP2 fullerene is a two-dimensional network of C_60_ cages connected by the out-of-plane vertical [2 + 2] cycloaddition bonds along the *a* direction and the in-plane [2 + 2] cycloaddition bonds along the *b* direction. The major difference between qTP1 and qTP2 is the absence of the vertical [2 + 2] cycloaddition bonds along *a* in the former. Regarding the qHP monolayer, the C_60_ cages form a hexagonal network through the similar planar [2 + 2] cycloaddition bonds along the *b* direction and C–C single bonds along the other two directions diagonal to the rectangular unit cell.

Figure 1 also shows the maps of the electron localization function (ELF) on the (010) plane. A high value of ELF indicates strong electron localization.^40−43^ As shown in Figure 1a, the covalent [2 + 2] bonds along *b* in qTP1 fullerene lead to high electron localization there (plane 1), whereas no bonds are formed between neighboring C_60_ cages along *a* (plane 2). In contrast, the vertical [2 + 2] bonds along *a* in qTP2 fullerene result in high electron localization between neighboring C_60_ cages, as demonstrated in plane 2 of Figure 1b. For qHP C_60_, the hexagonal network has higher electron localization in both directions, as one can see from Figure 1c. As a result, one can expect that the hexagonal networks should stabilize and strengthen the structure of qHP C_60_, making it slightly more stable than qTP2 C_60_ while being much more stable than qTP1 C_60_. However, as shown below, although the mechanical and dynamic stabilities are consistent with the ELF picture, the high electron localization in qHP C_60_ does not guarantee its thermodynamic stability.

To confirm the mechanical stability, the elastic constants are calculated by finite differences through finite distortions of the lattice.^44,45^ There are different ways to define the 2D elastic constants from the computed 3D coefficients.^17,46,47^ Here the 2D coefficients *C*_*ij*_^2D^ are renormalized by the *c* lattice constant (the spacing between 2D layers):^17,46^ i.e., *C*_*ij*_^2D^ = *c* × *C*_*ij*_^3D^. The obtained 2D elastic constants (including ionic relaxations) are given in Table 2 using the Voigt notation, 1 – *xx*, 2 – *yy*, 6 – *xy*, and the present results agree well with previous calculations.^10,11^ According to Born-Huang’s lattice dynamic theory,^48,49^ in monoclinic crystals (qTP1 and qHP with space groups *P*2/*m* and *Pc*, respectively), the mechanical stability criteria are given by
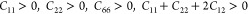
1

**Table 2:** Elastic Properties for qTP1, qTP2, and qHP C_60_, with the Elastic Constants *C*_*ij*_, Shear Moduli *G*^2D^, Layer Moduli γ, Young’s Moduli *Y*^2D^ in N/m, and Poisson’s Ratios ν (Dimensionless)^a^

phase	*C*_11_	*C*_22_	*C*_12_	*C*_66_ = *G*^2D^	γ	*Y*_*a*_^2D^	*Y*_*b*_^2D^	ν_*a*_	ν_*b*_
qTP1	5.4	123.7	–1.2	–0.2	31.7	5.4	123.5	–0.010	–0.225
	(2.5)	(121.3)							
qTP2	149.9	148.7	22.9	53.4	86.1	146.4	145.2	0.154	0.153
	(150.5)	(141.2)		(54.5)					
qHP	150.8	186.8	22.5	60.6	95.6	148.1	183.4	0.120	0.149
	(142.4)	(172.7)		(61.7)					

a The elastic constants *C*_11_, *C*_22_, and *C*_66_ calculated from the phonon speed of sound are also shown in parentheses for comparison.

In orthorhombic crystals (qTP2 C_60_ with space group *Pmmm*), the Born stability criteria have an extra requirement in addition to eq 1
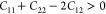
2

The elastic constants of qTP2 and qHP C_60_ satisfy their corresponding criteria, indicating that they are mechanically stable. Interestingly, the shear strength *G*^2D^ of qTP1 C_60_ is negative, demonstrating its low shear resistance. The 1D chains in qTP1 C_60_ are prone to bending under shear deformation, which may lead to a sliding of C_60_ chains and even lattice instability. In addition, *C*_11_ in qTP1 fullerene is more than 1 order of magnitude lower than *C*_22_. Such weak stiffness is correlated to the weak interchain bonding effect along *a*, as discussed above, and the weak dynamic stability, as will be demonstrated below. In contrast, *C*_11_ and *C*_22_ are nearly the same in qTP2 fullerene because of the similar [2 + 2] cycloaddition bonds along both *a* and *b*. For qHP fullerene, the elastic constants *C*_11_, *C*_22_, and *C*_66_ are the highest among the three phases, consistent with the high electron localization in the hexagonal networks that strengthens the crystal structure.

The strength of monolayer fullerene networks is obtained from the computed elastic constants, as summarized in Table 2. The layer modulus γ is the 2D equivalent to the bulk modulus, which measures the resistance to hydrostatic stretching in 2D materials.^50^ The layer moduli show an increasing trend from qTP1 to qTP2 and to qHP C_60_. The γ value for qTP2 C_60_ is more than twice that of qTP1 C_60_, while it is slightly lower than that of qHP C_60_, which concurs with the bonding structures of the three fullerene networks. In general, qTP2 and qHP C_60_ have comparable moduli and therefore similar hardness properties, whereas qTP1 C_60_ has less resilience to both shear and hydrostatic strains.

The anisotropy of strength is also investigated by calculating the Young’s modulus and Poisson’s ratio. In qTP1 fullerene, the Young’s modulus *Y*^2D^ along *a* is more than 22 times lower than that along *b*, indicating that qTP1 C_60_ is much less structurally rigid to elongations along *a*. In qTP2 C_60_, the Young’s moduli along *a* and *b* are nearly the same due to similar [2 + 2] cycloaddition bonds along both directions, showing that they have the same resilience to linear strain. Regarding the qHP monolayers, *Y*_*a*_^2D^ has a value 80% that of *Y*_*b*_^2D^, indicating slightly weaker stiffness of the C–C single bonds in the presence of strain along *a*. The Poisson’s ratio ν for qTP1 C_60_ is negative, i.e. the qTP1 fullerene monolayers expand laterally when stretched, and |ν_*a*_| is significantly lower than |ν_*b*_| because of much less bond stretching under uniaxial strain. Monolayer qTP2 C_60_ has a nearly isotropic ν of 0.153–0.154, while ν_*a*_ in qHP fullerene is slightly lower than ν_*b*_. These results indicate that qTP1 C_60_ is unable to withstand greater strains along *a* than those along *b*, which is the origin of its overall low strength.

To evaluate the dynamic stability of monolayer fullerene networks, lattice dynamic properties are calculated within the harmonic approximation based on density functional perturbation theory.^51−53^ The phonon spectra of all three phases are gathered in Figure 2. As shown in Figure 2a, the phonon dispersion of qTP1 C_60_ using the static lattice constants exhibits a small imaginary frequency (<0.6i THz) along the entire Γ–X high-symmetry line. An imaginary frequency indicates a decrease in potential energy when the atoms are displaced away from their equilibrium positions, corresponding to a nonrestorative force.^20^ Therefore, the imaginary frequency along Γ–X implies that monolayer qTP1 C_60_ can be split into individual 1D chains in the presence of interchain (out-of-plane) vibrations, demonstrating its weak dynamic stability along the *a* direction. There is a fourth mode at Γ with nearly zero frequency in qTP1 C_60_, which, sometimes known as the torsional acoustic mode, is a strong indication of the (quasi-)1D nature.^54,55^ The thermal expansion is included by computing the Gibbs free energy under the quasi-harmonic approximation,^56−58^ and the room-temperature lattice constants are given in Table 1. At 300 K, the imaginary mode in qTP1 C_60_ remains along Γ–X, though the imaginary frequency becomes smaller (<0.2i THz). In contrast, qTP2 and qHP fullerenes are dynamically more stable, as there is no imaginary mode in Figure 2b,c using both the static and room-temperature lattice constants, indicating that these structures are a local minimum on the potential energy surface and the atoms vibrate harmonically around their equilibrium positions.

**Figure 2:**
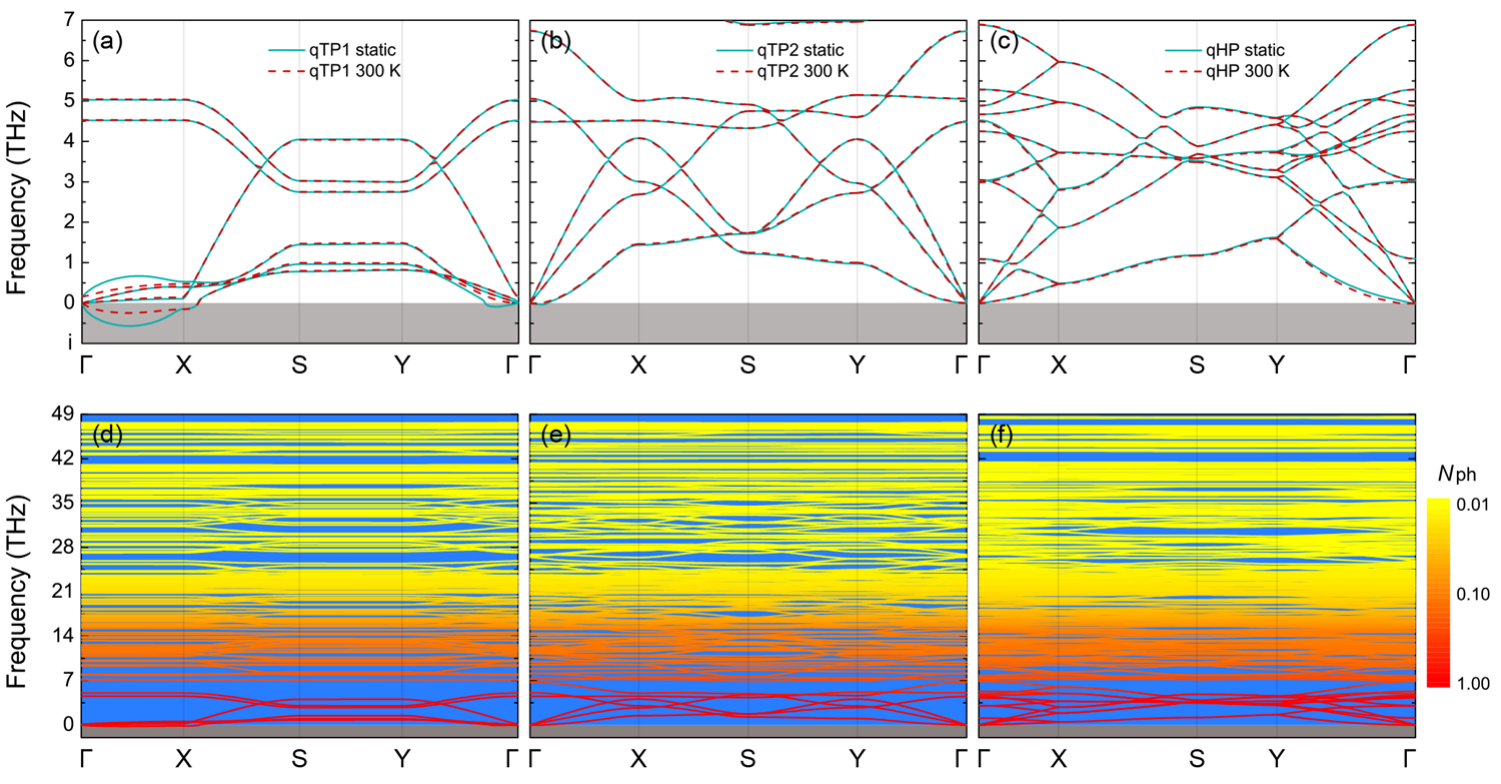
Low-frequency phonons of (a) qTP1, (b) qTP2, and (c) qHP C_60_ using the static and room-temperature lattice constants. Entire phonon spectra for (d) qTP1, (e) qTP2, and (f) qHP C_60_ using the room-temperature lattice constants, with the phonon occupation number *N*_ph_ being determined from the Bose–Einstein distribution function at 300 K.

From the phonon speed of sound, the elastic constants *C*_11_, *C*_22_, and *C*_66_ can be calculated^59^ (for details on the phonon group velocity, see the Supporting Information). As shown in Table 2, the calculated elastic constants are in reasonably good agreement with those computed from the finite difference method.^44,45^ Moreover, qHP fullerene has the highest speed of sound along *b* and the highest phonon frequency with four phonon branches higher than 48.4 THz throughout the entire Brillouin zone, whereas the highest phonon frequencies in qTP1 and qTP2 fullerene are lower than 47.7 THz, which is in line with the high mechanical strength in qHP C_60_.

To clarify the thermodynamic stability of C_60_ monolayers, the cohesive energy is calculated, as given in Table 1. The resulting *E*_c_ of qTP2 fullerene is 0.5 meV/atom (30 meV per formula unit) lower than that of qTP1 and 12.2 meV/atom (732 meV per formula unit) lower than that of qHP, suggesting its thermodynamic stability. However, because the energy difference between the qTP1 and qTP2 monolayers is quite small, phonons can play an important role in determining the thermodynamic stability at both 0 K and finite temperatures.^21−24^ The contribution of phonons can be examined by calculating the Gibbs free energy *F* ^60−62^
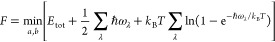
3where  finds a unique minimum value in the brackets by changing the lattice constants *a* and *b* to include thermal expansion, *E*_tot_ is the total energy of the crystal, *ℏ* is the reduced Planck constant, ω_λ_ is the phonon frequency at mode λ, *k*_B_ is the Boltzmann constant, and *T* is the temperature. The second term in eq 3 is temperature-independent, corresponding to the zero point energy (ZPE) of phonons, and the last term refers to the thermally excited population of phonons, as demonstrated by the Bose–Einstein distribution *N*_ph_ at 300 K in Figure 2d–f.

To quantify the relative thermodynamic stability at finite temperatures, the difference in Gibbs free energy Δ*F* with respect to the free energy of monolayer qTP2 C_60_ is plotted as a function of temperature *T* for all three phases, as illustrated in Figure 3. With increasing temperature, the free energy of qTP1 C_60_ drops faster than that of qTP2 C_60_ due to its smaller vibrational frequencies. According to eq 3, smaller vibrational frequencies give rise to lower free energy and higher entropy (for details on the phonon density of states and entropy, see the Supporting Information), which is similar to the case in α- and β-tin.^21,22^ At 150 K, monolayer qTP1 C_60_ becomes thermodynamically more stable than the other two phases. At 300 K, the free energy of qTP1 C_60_ lies 47 meV per formula unit (meV/fu) below that of qTP2 C_60_. At higher temperatures, the difference becomes even larger, further stabilizing the qTP1 structure from a thermodynamic perspective.

**Figure 3:**
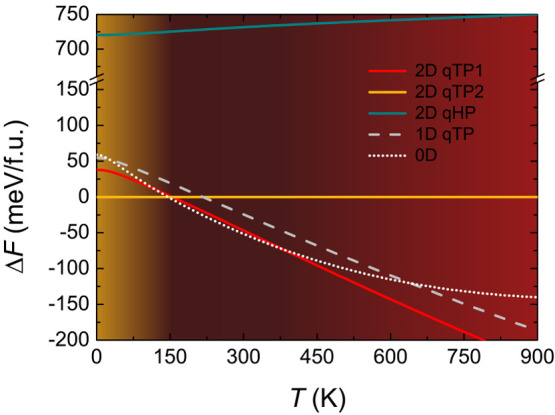
Relative thermodynamic stabilities of monolayer fullerene networks, a one-dimensional fullerene chain, and a zero-dimensional fullerene molecule, with the Gibbs free energy *F* of monolayer qTP2 C_60_ set to zero to compare the relative stabilities.

To further explore the thermodynamic stability of C_60_ in different dimensions, the monolayer qTP1 network is further isolated into a 1D qTP C_60_ chain and a 0D C_60_ molecule (for details about 1D and 3D C_60_, see the Supporting Information). The cohesive energies *E*_c_ of 1D qTP C_60_ and 0D C_60_ are given in Table 1. Interestingly, *E*_c_ of the 1D qTP C_60_ chain is higher than those of monolayer qTP1 and qTP2 C_60_ network by merely 0.3 and 0.8 meV/atom, respectively (18 and 48 meV/fu), whereas it is 11.4 meV/atom (684 meV/fu) lower than *E*_c_ of 2D qHP C_60_. The difference in *E*_c_ between the 1D qTP C_60_ chain and the monolayer qTP1 C_60_ network is even lower than the thermal fluctuation energy *k*_B_*T* at room temperature (26 meV), implying that 2D qTP1 C_60_ can be transformed into 1D chains in the presence of thermal fluctuations. Taking the finite temperature effects into account, the Gibbs free energy of the 1D qTP C_60_ chain and the Helmholtz free energy of the 0D C_60_ molecule are shown in Figure 3 as a function of temperature. The Gibbs free energy of the 1D qTP C_60_ chain is higher than that of 2D qTP1 C_60_ in the entire temperature range (0–900 K), and their free energy difference is 22 meV/fu at 300 K. On the other hand, the free energy of the 1D qTP C_60_ chain becomes lower than that of 2D qTP2 C_60_ at temperatures above 220 K. Most interestingly, the free energy of the 0D C_60_ molecule drops faster than those for all the other phases below room temperature and becomes lower than those of 2D qTP1 and qTP2 C_60_ at 120 and 150 K, respectively. However, the free energy of 2D qTP1 C_60_ decreases the fastest above room temperature, and consequently 2D qTP1 C_60_ is energetically more favored than all the other phases at temperatures above 380 K. As a result, monolayer qTP2 C_60_ is thermodynamically the most stable at temperatures below 150 K, the 0D C_60_ molecule has the lowest energy for temperatures between 150 and 380 K, and 2D qTP1 C_60_ is thermodynamically favored above 380 K.

Looking back at the calculated mechanical properties and stabilities, they seem in line with the experimental findings. It has been reported that fullerene monolayers can only be isolated experimentally for the honeycomb structure qHP, whereas the obtained rectangular structure qTP is few-layered.^1^ Although qTP2 C_60_ is thermodynamically favored over qTP1 C_60_ at low temperatures, clearly qTP1 C_60_ is thermodynamically more stable than the other two phases at all temperatures above 150 K. However, the thermodynamic stability of qTP1 C_60_ does not guarantee high dynamic stability in the presence of interchain (out-of-plane) vibrations perpendicular to the quasi-1D chains. In addition, the low moduli and strength of qTP1 C_60_ originating from the chain crystal structures, in addition to its low shear resistance, indicate that qTP1 C_60_ cannot be intrinsically resilient under deformation. Moreover, monolayer qTP1 C_60_ is thermodynamically less stable than the 0D C_60_ molecule for temperatures between 120 and 380 K. These results indicate the plausibility that the monolayer qTP1 fullerene network can be further split into individual 1D chains or 0D molecules by thermal fluctuations, interchain acoustic vibrations, or external strains. In contrast, qHP C_60_ is both dynamically and mechanically more stable with respect to qTP1 C_60_. Therefore, monolayer polymeric C_60_ has so far only been exfoliated from the quasi-hexagonal bulk single crystals. These results indicate that a systematic analysis of mechanical, dynamic, and thermodynamic stabilities is necessary to rationalize the experimental data.

In conclusion, I carry out first-principles calculations to evaluate the mechanical, dynamic, and thermodynamic stabilities of qTP1, qTP2, and qHP C_60_ monolayers, which have been so far believed to be stable. The electron localization analysis reveals that the low mechanical and dynamic stabilities in qTP1 fullerene are associated with the lack of C–C bonds connecting the adjacent C_60_ chains, which also limits its achievable strength. Monolayer qTP2 C_60_ is thermodynamically more stable at temperatures below 150 K, while thermally populated phonons hinder its thermodynamic stability with increasing temperature. The relatively high moduli of qHP fullerene indicate that it has a high strength because of the closely packed hexagonal fullerene network linked through both [2 + 2] cycloaddition bonds and C–C single bonds. This, in combination with the phonon stability, endows monolayer qHP C_60_ with high stability and strength.

## Supporting Information Available

Computational details, phonon group velocity, phonon density of states and entropy, 1D qTP C_60_ chains, and relative thermodynamic stabilities between 3D, 2D, 1D, and 0D C_60_ (PDF)
