# Supplementary material for: Stability and Strength of Monolayer Polymeric C60
Source: Nano Lett. 2023 Jan 11;23(2):652–8. doi: 10.1021/acs.nanolett.2c04497 (PMC9881160; doi:10.1021/acs.nanolett.2c04497)
Supplement: Supplementary file 1 — nl2c04497_si_001.pdf [file nl2c04497_si_001.pdf]

Supporting Information for
"Stability and strength of monolayer polymeric C_{60}"

Bo Peng[1,*]

[1] *Theory of Condensed Matter Group, Cavendish Laboratory,*
University of Cambridge, J. J. Thomson Avenue,
Cambridge CB3 0HE, United Kingdom

(Dated: January 6, 2023)

A. Computational details

Density functional theory calculations are performed using the Vienna *ab-initio* simulation package (VASP) [1,2]. The projector augmented wave (PAW) potential is used with C $2s^22p^2$ valence states [3,4], under the generalized gradient approximation (GGA) with the Perdew-Burke-Ernzerhof parameterization revised for solids (PBEsol) as the exchange-correlation functional [5]. For structural relaxation, a plane-wave cutoff of 800 eV is used with a **k**-mesh of 5×5 and 3×5 for qTP and qHP C_{60} respectively, until the energy difference between successive steps is below 10^{-6} eV and the forces on the atoms are below 10^{-2} eV/Å. A vacuum spacing larger than 17 Å is used to eliminate interactions between neighboring unit cells along the c direction.

The elastic tensor coefficients (including ionic relaxations) are calculated using the finite differences method [6,7], and then renormalized by the c lattice constant ($C_{ij}^{\text{2D}} = c \times C_{ij}^{\text{3D}}$) [8,9].

The phonon spectra are calculated under the harmonic approximation based on density functional perturbation theory [10–12] using the PHONOPY code [13,14]. The supercell size is 2×2 for qTP C_{60} with an electronic **k**-point grid of (Γ-centered) 2×2, which is well converged, as shown in Fig. 1. The supercell size is 1×2 for qHP C_{60} because the lattice constant a is nearly two times the b. The thermodynamic properties are calculated using a phonon **q**-mesh of 91×91 for both qTP and qHP C_{60} monolayers.

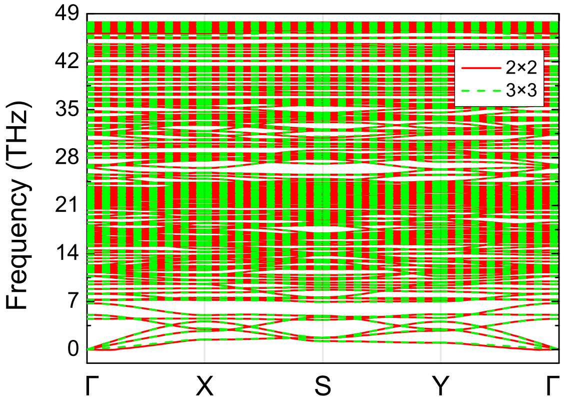

FIG. 1. Phonon spectra of qTP2 C_{60} calculated from 2×2 and 3×3 supercells.

The role of thermal expansion is investigated under the quasi-harmonic approximation. The Helmholtz free energy is calculated for anisotropically contracted and expanded lattice with a strain step of 0.4%, and at least 25 configurations are included for each phase when

fitting the Gibbs free energy for a temperature step of 10 K between 0−900 K.

B. Phonon group velocity

Figure 2 shows the calculated phonon group velocity v_g in 2D qTP2 and qHP C_{60}. The elastic constants can be estimated from the phonon speed of sound, i.e. phonon group velocity v_g near Γ [15]

$$C_{11} = \rho^{2D} \times \left(v_x^{LA}\right)^2, \tag{1}$$

$$C_{22} = \rho^{2D} \times \left(v_y^{LA}\right)^2, \tag{2}$$

$$C_{66} = \rho^{2D} \times \left(v_x^{TA}\right)^2, \tag{3}$$

where ρ^{2D} is the mass density in 2D and $v_{x/y}^{LA/TA}$ is the speed of sound for the longitudinal or transverse acoustic mode (LA/TA) along x/y. The computed elastic constants are listed in Table 2 in the main text.

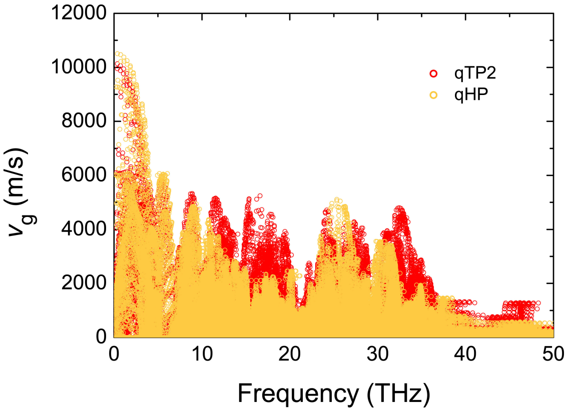

FIG. 2. Phonon group velocity in 2D qTP2 and qHP C_{60}.

C. Phonon density of states and entropy

With increasing temperature, the free energy F of qTP1 C_{60} drops faster than that of qTP2 C_{60} due to their smaller vibrational frequencies. As shown in Fig. 3(a), the cumulative phonon density of states (phDOS) of qTP1 fullerene is much larger than that of qTP2 and qHP fullerene at frequencies below 7 THz. The low phonon frequencies in qTP1 C_{60} give rise to higher entropy S in Fig. 3(b), especially at temperatures below 50 K. Consequently,

the Helmholtz free energy of qTP1 C_{60} drops faster because of its higher entropy S, which gives the curve of the free energy F as a function of temperature T [16,17]

$$S = -\partial F/\partial T. \tag{4}$$

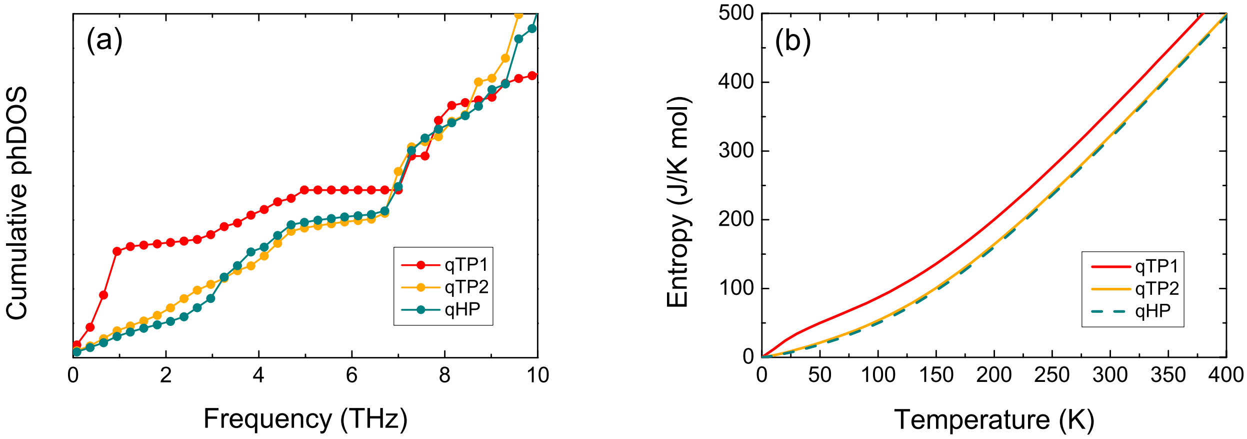

FIG. 3. (a) Cumulative phDOS and (b) phonon entropy of 2D qTP1, qTP2 and qHP C_{60}.

D. 1D qTP C_{60} chains

For structural relaxation of 1D qTP C_{60} chains, a plane-wave cutoff of 800 eV is used with a **k**-mesh of 5 along the chain direction, until the energy difference between successive steps is below 10^{-6} eV and the forces on the atoms are below 10^{-2} eV/Å. A vacuum spacing larger than 20 Å is used to eliminate interactions between neighboring unit cells along the interchain directions. The top view of the crystal structure of 1D qTP C_{60} chain is shown in Fig. 4(a). The strong covalent [2+2] bonds along a leads to high electron localization function (ELF) values. The band structures in Fig. 4(b) are calculated using screened hybrid functional HSE06 [18–20], with a direct band gap of 1.81 eV at the X high-symmetry point.

The interatomic force constants are calculated under the harmonic approximation based on density functional perturbation theory [10–12] using the PHONOPY code [13,14], with a supercell of four qTP C_{60} molecules along the a direction. No imaginary mode is observed in Fig. 4(c), showing its dynamic stability. The extra zero-frequency mode at Γ other than the three acoustic modes is the typical torsional acoustic mode in 1D materials [21,22]. For thermal

expansion calculations, the Helmholtz free energy is calculated for 8 uniaxially contracted and expanded configurations with a strain step of 0.2% to fit the Gibbs free energy.

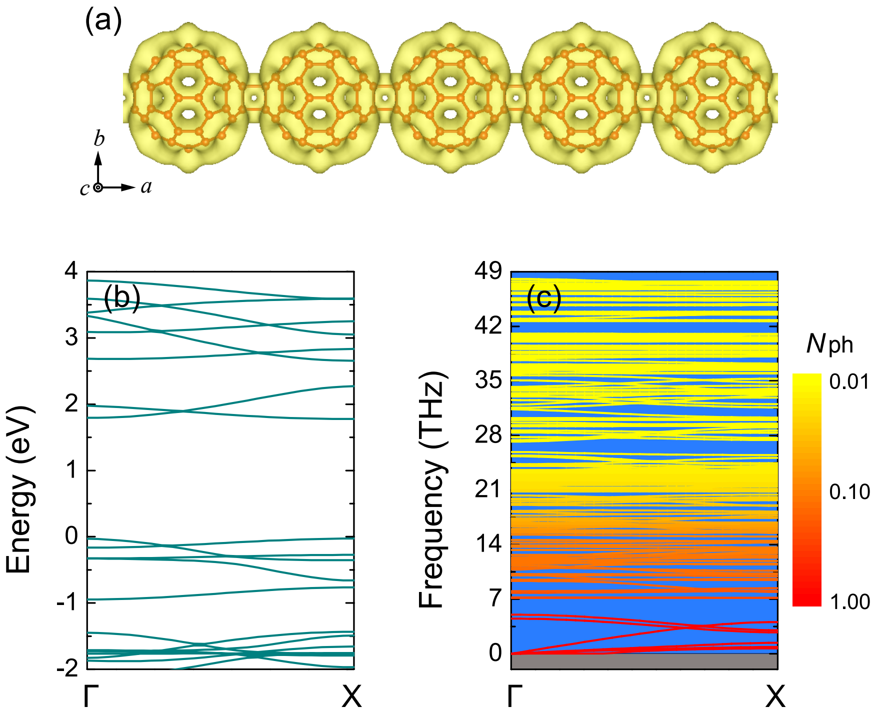

FIG. 4. (a) ELF of 1D qTP C_{60} chain. The default isosurface level in VESTA [23] is used. (b) HSE06 band structures of 1D qTP C_{60}. (c) Phonon dispersion of 1D qTP C_{60} with the phonon occupation number N_{ph} determined from the Bose-Einstein distribution function at 300 K.

E. Relative thermodynamic stability between 3D, 2D, 1D and 0D C_{60}

For structural relaxation of bulk polymeric C_{60}, a plane-wave cutoff of 800 eV is used with a **k**-mesh of $5 \times 5 \times 3$ and $3 \times 5 \times 3$ for qTP and qHP C_{60} respectively, until the energy difference between successive steps is below 10^{-6} eV and the forces on the atoms are below 10^{-2} eV/Å. The cohesive energy E_{c} for bulk single crystal polymeric C_{60} is calculated and compared with their low-dimensional counterparts. As shown in Fig. 5, both 3D qTP1 and qHP C_{60} are thermodynamically more stable than their 2D counterparts, whereas 3D qTP2 C_{60} is energetically less favored than 2D qTP2 C_{60}. Among all the bulk single crystal polymeric C_{60}, 3D qTP1 C_{60} has the lowest E_{c} while 3D qHP C_{60} has the highest E_{c}.

To include the contribution of phonons, the Helmholtz free energy of 3D, 2D, 1D and 0D C_{60} is calculated using the static lattice constants. The supercell size is $2 \times 2 \times 1$ and

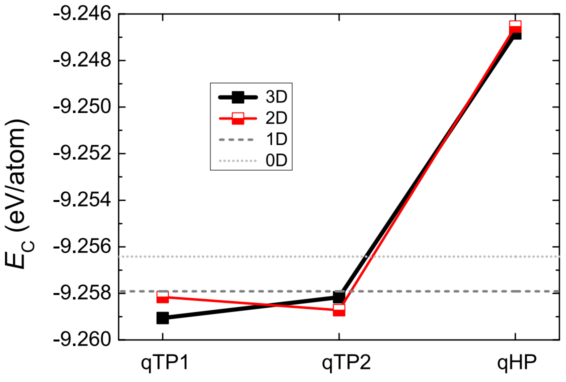

FIG. 5. Cohesive energy of bulk single crystal polymeric C_{60} and their low-dimensional counterparts.

$1 \times 2 \times 1$ for 3D qTP and qHP C_{60} respectively. Figure 6 shows the Helmholtz free energy of 3D, 2D and 1D qTP C_{60} as a function of temperature, with the free energy of monolayer qTP2 C_{60} set to zero to compare the relative stability. Interestingly, bulk qTP1 C_{60} has the lowest free energy in the entire temperature range ($0 - 1000$ K), whereas bulk qTP2 C_{60} has the highest free energy above 50 K. It should be noted that, different from the Gibbs free energy in the main text that includes the contribution of thermal expansion, for all the structural phases in Fig. 6 thermal expansion is not included because the computational cost for thermal expansion in anisotropic bulk materials is much higher than that in their 2D counterparts.

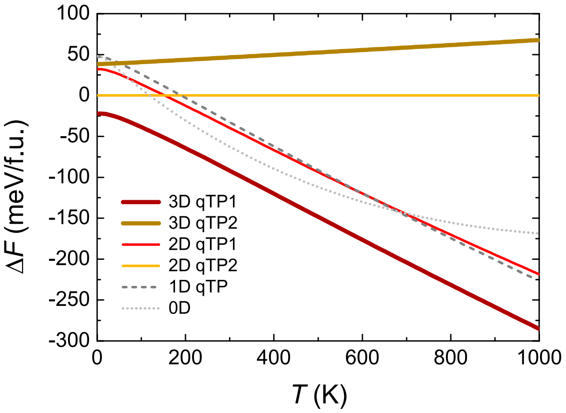

FIG. 6. Relative thermodynamic stability of bulk polymeric C_{60}, monolayer fullerene networks, one-dimensional fullerene chain and zero-dimensional fullerene molecule.

* bp432@cam.ac.uk

[1] G. Kresse and J. Furthmüller, "Efficient iterative schemes for *ab initio* total-energy calculations using a plane-wave basis set," Phys. Rev. B **54**, 11169–11186 (1996).

[2] G. Kresse and J. Furthmüller, "Efficiency of *ab-initio* total energy calculations for metals and semiconductors using a plane-wave basis set," Computational Materials Science **6**, 15 – 50 (1996).

[3] P. E. Blöchl, "Projector augmented-wave method," Phys. Rev. B **50**, 17953–17979 (1994).

[4] G. Kresse and D. Joubert, "From ultrasoft pseudopotentials to the projector augmented-wave method," Phys. Rev. B **59**, 1758–1775 (1999).

[5] John P. Perdew, Adrienn Ruzsinszky, Gábor I. Csonka, Oleg A. Vydrov, Gustavo E. Scuseria, Lucian A. Constantin, Xiaolan Zhou, and Kieron Burke, "Restoring the Density-Gradient Expansion for Exchange in Solids and Surfaces," Phys. Rev. Lett. **100**, 136406 (2008).

[6] Yvon Le Page and Paul Saxe, "Symmetry-general least-squares extraction of elastic data for strained materials from *ab initio* calculations of stress," Phys. Rev. B **65**, 104104 (2002).

[7] Xifan Wu, David Vanderbilt, and D. R. Hamann, "Systematic treatment of displacements, strains, and electric fields in density-functional perturbation theory," Phys. Rev. B **72**, 035105 (2005).

[8] Michael N. Blonsky, Houlong L. Zhuang, Arunima K. Singh, and Richard G. Hennig, "Ab initio prediction of piezoelectricity in two-dimensional materials," ACS Nano **9**, 9885–9891 (2015).

[9] Bo Peng, Hao Zhang, Hezhu Shao, Zeyu Ning, Yuanfeng Xu, Gang Ni, Hongliang Lu, David Wei Zhang, and Heyuan Zhu, "Stability and strength of atomically thin borophene from first principles calculations," Materials Research Letters **5**, 399–407 (2017).

[10] Stefano Baroni, Stefano de Gironcoli, Andrea Dal Corso, and Paolo Giannozzi, "Phonons and related crystal properties from density-functional perturbation theory," Rev. Mod. Phys. **73**, 515–562 (2001).

[11] Xavier Gonze, "Perturbation expansion of variational principles at arbitrary order," Phys. Rev. A **52**, 1086–1095 (1995).

[12] Xavier Gonze, "Adiabatic density-functional perturbation theory," Phys. Rev. A **52**, 1096–1114 (1995).

[13] Atsushi Togo, Fumiyasu Oba, and Isao Tanaka, "First-principles calculations of the ferroelastic transition between rutile-type and $CaCl_2$-type SiO_2 at high pressures," Phys. Rev. B **78**, 134106 (2008).

[14] Atsushi Togo and Isao Tanaka, "First principles phonon calculations in materials science," Scripta Materialia **108**, 1–5 (2015).

[15] Jing Feng, Bing Xiao, Rong Zhou, Wei Pan, and David R. Clarke, "Anisotropic elastic and thermal properties of the double perovskite slab-rock salt layer $Ln_2SrAl_2O_7$ (Ln = La, Nd, Sm, Eu, Gd or Dy) natural superlattice structure," Acta Materialia **60**, 3380–3392 (2012).

[16] Martin T. Dove, Introduction to Lattice Dynamics (Cambridge University Press, 1993).

[17] Pasquale Pavone, Stefano Baroni, and Stefano de Gironcoli, "$\alpha \leftrightarrow \beta$ phase transition in tin: A theoretical study based on density-functional perturbation theory," Phys. Rev. B **57**, 10421–10423 (1998).

[18] Jochen Heyd, Gustavo E. Scuseria, and Matthias Ernzerhof, "Hybrid functionals based on a screened Coulomb potential," J. Chem. Phys. **118**, 8207 (2003).

[19] Jochen Heyd, Gustavo E. Scuseria, and Matthias Ernzerhof, "Erratum: 'Hybrid functionals based on a screened Coulomb potential' [J. Chem. Phys.118, 8207 (2003)]," J. Chem. Phys. **124**, 219906 (2006).

[20] Juan E. Peralta, Jochen Heyd, Gustavo E. Scuseria, and Richard L. Martin, "Spin-orbit splittings and energy band gaps calculated with the Heyd-Scuseria-Ernzerhof screened hybrid functional," Phys. Rev. B **74**, 073101 (2006).

[21] Bo Peng, Ke Xu, Hao Zhang, Zeyu Ning, Hezhu Shao, Gang Ni, Jing Li, Yongyuan Zhu, Heyuan Zhu, and Costas M. Soukoulis, "1D SbSeI, SbSI, and SbSBr With High Stability and Novel Properties for Microelectronic, Optoelectronic, and Thermoelectric Applications," Adv. Theory Simul. **1**, 1700005 (2018).

[22] Gunnar F. Lange, Adrien Bouhon, Bartomeu Monserrat, and Robert-Jan Slager, "Topological continuum charges of acoustic phonons in two dimensions and the nambu-goldstone theorem," Phys. Rev. B **105**, 064301 (2022).

[23] Koichi Momma and Fujio Izumi, "Vesta 3 for three-dimensional visualization of crystal, volumetric and morphology data," Journal of Applied Crystallography **44**, 1272–1276 (2011).
